# Quality of life and chronic musculoskeletal disorders: a multifactorial study of the impacts on the health of office workers

**DOI:** 10.47626/1679-4435-2025-1396

**Published:** 2025-11-04

**Authors:** Beatriz Oliveira Vieira, Bernardino Geraldo Alves Souto, Claudia Aparecida Stefane

**Affiliations:** 1 Universidade Federal de São Carlos, Departamento de Medicina, São Carlos, SP, Brazil

**Keywords:** chronic pain, ergonomics, quality of life, occupational health., dor crônica, ergonomia, qualidade de vida, saúde ocupacional.

## Abstract

**Introduction:**

Office workers are at higher risk of musculoskeletal disorders due to prolonged sitting and repetitive movements, impacting their quality of life and generating costs for employers, healthcare systems, and social security.

**Objectives:**

To investigate the presence of chronic musculoskeletal disorders and their correlation with quality of life among employees at a federal higher education institution.

**Methods:**

A descriptive study based on an approved ethics committee database. Participants included 256 workers who worked 20 hours/week in office settings and provided sociodemographic data, chronic musculoskeletal disorder assessments (Nordic Musculoskeletal Questionnaire), and quality of life evaluations (WHOQOL-Bref). Quality of life scores were compared using Pearson’s correlation test, with a significance level of 95% (p < 0.05).

**Results:**

The median age was 39 years, with a female majority (66.3%), married (62.3%), and holding a college degree (98%). Of the total, 186 (72.6%) reported chronic musculoskeletal disorders, primarily in the neck and lower back. Wrist-hand disorders were associated with worse overall quality of life scores, as well as physical and environmental domains, while lower back disorders correlated with poorer social domain scores.

**Conclusions:**

Despite being young adults, most participants had disorders in multiple body regions; however, only wrist-hand symptoms were significantly correlated with worse overall quality of life. The adoption of health education measures is recommended to mitigate and prevent these conditions.

## Abstract (pt)

**Introdução:**

Atuantes em ambiente de escritório têm maiores riscos de distúrbios osteomusculares devido à postura sentada prolongada e movimentos repetitivos, impactando na qualidade de vida e gerando custos para empresa, sistemas de saúde e previdenciário.

**Objetivos:**

Investigar a presença de distúrbios osteomusculares crônicos e a correlação com a qualidade de vida em servidores de uma instituição federal de ensino superior.

**Métodos:**

Estudo descritivo baseado em banco de dados de pesquisas aprovadas pelo comitê de ética. Participaram 256 trabalhadores, que trabalhavam 20 horas semanais em escritório e que tinham os dados sociodemográficos de distúrbios osteomusculares crônicos (Questionário Nórdico de Distúrbios Osteomusculares) e de qualidade de vida (World Health Organization Quality of Life). A comparação dos escores de qualidade de vida foi realizada pelo teste de correlação de Pearson, com nível de significância de 95% (p < 0,05).

**Resultados:**

A mediana de idade foi 39 anos, maioria feminina (66,3%), casada (62,3%) e com ensino superior completo (98%). Do total, 186 (72,6%) referiram distúrbios osteomusculares crônicos, principalmente nas regiões do pescoço e parte inferior das costas. Os distúrbios no punho-mão se relacionaram com pior escore de qualidade de vida global e nos domínios físico e ambiental; e os da região lombar, com pior no domínio social.

**Conclusões:**

Apesar de jovens adultos, a maioria estava adoecida em múltiplas regiões; entretanto, a correlação dos distúrbios com pior qualidade de vida global ocorreu somente com sintomas no punho-mão. Sugere-se a adoção de medidas de educação em saúde para mitigar e prevenir o adoecimento.

## INTRODUCTION

Pain is a major public health problem that is underdiagnosed and undertreated.^1^ In Brazil, according to the Brazilian Society of Orthopedics and Traumatology, limited awareness of the need for proper assessment contributes to this gap, and pain affects about 37% of Brazilians^2^. In addition, the Epidemiology of Pain Study from the University of São Paulo’s School of Public Health found that over 45% of people with back pain lasting longer than 3 months do not seek appropriate treatment.^2^

Office work environments can contribute to the development of chronic musculoskeletal disorders (CMSDs).^1^ Poor posture, prolonged sitting, repetitive tasks, and limited movement exacerbate injuries and pain.^3^,^4^ Organizational factors (eg, rigid rules, inflexible hierarchies, inefficient communication, and centralized decision-making) may further increase physical and mental overload.^5^

In 2019, Brazil’s Special Secretariat for Social Security and Labor recorded roughly 39,000 work leave episodes due to work-related musculoskeletal disorders (WRMDs) or repetitive strain injury (RSI).^6^ These conditions negatively affect workers’ lives and also burden employers and public health and social security systems.

These disorders involve muscles, tendons, joints, nerves, and ligaments, and manifest through signs and symptoms such as pain and reduced range of motion or muscle strength.^7^ Pain is the most prevalent symptom in WRMD or RSI.

Pain may be acute or chronic. Acute pain is a physiological response to short-term noxious stimuli, whereas chronic pain persists beyond normal tissue healing time and is considered a disease.^8^ Chronic pain may negatively impact quality of life (QoL) and be linked to sleep disturbances, anxiety, and depression.^4^,^7^,^9^

QoL is defined by the World Health Organization (WHO) as an individual’s perception of their position in life within the context of their culture, encompassing physical, mental, social, and environmental domains.^10^

Based on the premise that physical factors affect QoL, our hypothesis is that pain may undermine autonomy by reducing physical and functional capacities. Accordingly, this study aimed to investigate the presence of CMSDs and their correlation with QoL among civil servants at a federal higher education institution.

## METHODS

This was a descriptive, cross-sectional study using aggregated, anonymized secondary data collected between 2018 and 2024 and provided by the Worker Health Study Group (GEST, in Portuguese). Data access can be requested from https://www.instagram.com/gpe_saudecoletiva?igsh=MWoxajR1NGc5OHY3Yg==.

The sample comprised civil servants from a federal higher education institution who performed office activities for at least 20 hours per week. These roles are predominantly technology-based and involve the use of computers and accessories. Inclusion criteria were age 18 years or older and working in an office at least 20 hours/week; pregnancy was an exclusion criterion. Variables included age, sex, education level, marital status, sitting time, screen time (eg, computers, mobile phones, and television), presence of CMSDs, and QoL scores. Because the dataset was aggregated and de-identified, institutional ethics review was not required, in accordance with Article 1, sole paragraph, items II and V of National Health Council Resolution 510 (04/07/2016).

QoL data were obtained with the World Health Organization Quality of Life assessment, short form (WHOQOL-BREF), developed by the WHO and validated in Portuguese by Fleck (2000).^11^ The instrument assesses QoL in adults (past 2 weeks) using 26 items across four domains: physical, psychological, environment, and social relationships. Items are scored on a 5-point Likert scale; domain scores range from 0 to 100, where 0 indicates worse QoL and 100 indicates better QoL.

CMSDs were assessed with a question from the Nordic Musculoskeletal Questionnaire, which captures symptoms over the past year in nine body regions (neck, shoulders, upper back, elbows, wrists/hands, lower back, hips/thighs, knees, and ankles/feet). Responses are dichotomous (yes/no). The instrument was translated and validated in Portuguese by Barros & Alexandre.^9^

Age, sitting time, and screen time were summarized with measures of central tendency and spread. Sociodemographic variables were displayed in simple tabulations by category. The count of symptomatic body regions was treated as a categorical variable, with each specific count shown in simple tabulation. A body-map diagram was used to display the regions affected and the proportion of the sample reporting each region as symptomatic.

QoL scores were categorized as global and by domain (physical, psychological, social, and environmental) and presented in simple tabulations. Pearson correlations were then calculated between each QoL domain and i) each affected region and ii) the number of affected regions. Correlations were considered significant at p < 0.05.

Data were processed using IBM SPSS Statistics for Windows, version 20.0 (IBM Corp., Armonk, N.Y., USA), jamovi (https://www.jamovi.org/), Epi Info (https://www.cdc.gov/epiinfo/por/pt_index.html), and the Statistics Kingdom online calculator (https://www.statskingdom.com/121proportion_normal2.html).

## RESULTS

The dataset comprised 256 individuals. Ages ranged from 25 to 71 years, with a median of 39 years; most participants (57.54%) were at or below the median age. On average, participants reported 44 hours per week of screen time and 47 hours per week spent sitting. Most were women (66.3%), married (62.3%), and college educated (98%). The prevalence of musculoskeletal symptoms was 18% higher among women than men (78% in women vs 66% in men; p = 0.041).

Of the 256 participants, 186 (72.6%) reported at least 1 CMSD symptom. Of these, 117 had up to 3 symptomatic body regions (Table 1).

**Table 1:** Number of individuals by count of chronically symptomatic body regions

Number of regions affected	n	%	Cumulative %
1	29	11.51	37.70
2	46	18.25	55.95
3	42	16.67	72.62
4	24	9.52	82.14
5	16	6.35	88.49
6	14	5.56	94.05
7	7	2.78	96.83
8	5	1.98	98.81
9	3	1.19	100.00
Total	186	100.00	100.00

**Figure 1:**
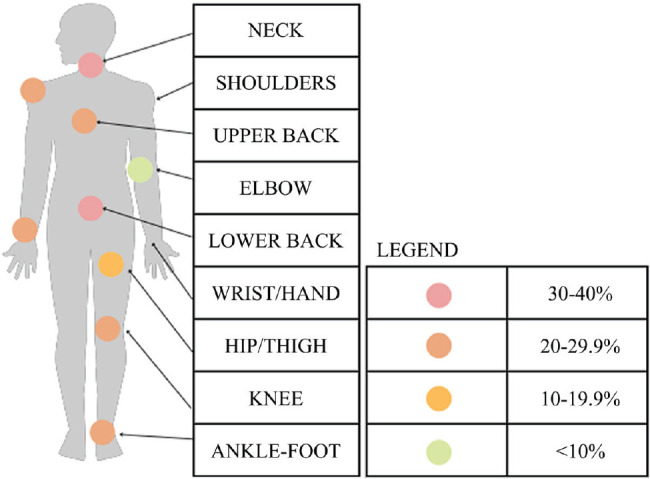
Percentage of individuals with chronic musculoskeletal symptoms, by body region.

Weekly screen time and time spent sitting were the only variables positively correlated with the presence of CMSDs (p < 0.05), and they were positively correlated with each other. Female sex remained an independent predictor of CMSDs (p = 0.026). The most commonly affected regions were the cervical and lumbar areas (Figure 1).

With respect to QoL, the greatest impairment was in the psychological and physical domains (Table 2).

**Table 2:** Quality of life: global and domain scores

Variable	Maximum score = 100% Mean score (%)
Global	67
Physical domain	66
Psychological domain	64
Social domain	70
Environmental domain	69

For both global and domain-specific QoL, a higher number of affected regions was associated with lower global QoL. Although the number of affected regions was not significantly correlated with the psychological domain, it was inversely correlated with the other domains. At the same time, all QoL domains were directly related to global QoL - i.e., worse scores in any domain correlated with worse global QoL (Table 3).

**Table 3:** Correlation between global and domain-specific quality of life (QoL), and with the number of chronically affected regions

QoL	Statistic	No. of regions affected	Global QoL
Global	Correlation	-0.281	
	p-value	< 0.001	
By domain			
Physical	Correlation	-0.389	0.848
	p-value	< 0.001	< 0.001
Psychological	Correlation	0.124	0.558
	p-value	0.048	< 0.001
Social	Correlation	-0.293	0.916
	p-value	< 0.001	< 0.001
Environmental	Correlation	-0.216	0.910
	p-value	< 0.001	< 0.001

When QoL was analyzed by affected region, chronic wrist-hand symptoms showed the strongest associations with worse global QoL and with poorer physical and environmental domain scores. Chronic lumbar involvement was most strongly associated with worse QoL in the social domain (Table 4).

**Table 4:** Pearson correlation between chronically affected region and global and domain-specific quality of life (QoL)

Affected region	Stastic	Global QoL	QoL domains			
			Physical	Psychological	Social	Environmental
Neck/cervical	Correlation	-0.096	-0.150	0.002	-0.078	-0.053
	p-value	0.127	0.016	0.977	0.212	0.397
Shoulder	Correlation	-0.155	-0.207	0.055	-0.152	-0.125
	p-value	0.013	< 0.001	0.381	0.015	0.047
Elbow	Correlation	-0.132	-0.170	0.027	-0.126	-0.107
	p-value	0.034	0.006	0.666	0.043	0.088
Wrist-hand	Correlation	-0.188	-0.253	0.026	-0.171	-0.142
	p-value	0.003	< 0.001	0.680	0.006	0.023
Upper back	Correlation	-0.084	-0.107	0.019	-0.107	-0.032
	p-value	0.182	0.087	0.763	0.086	0.612
Lumbar	Correlation	-0.175	-0.221	0.013	-0.175	-0.120
	p-value	0.005	< 0.001	0.840	0.005	0.054
Hip-thigh	Correlation	-0.130	-0.212	0.048	-0.118	-0.072
	p-value	0.038	< 0.001	0.441	0.060	0.253
Knee	Correlation	-0.104	-0.149	0.023	-0.100	-0.063
	p-value	0.096	0.017	0.715	0.112	0.317
Ankle-foot	Correlation	-0.086	-0.176	-0.027	-0.022	-0.042
	p-value	0.172	0.005	0.662	0.722	0.507

## DISCUSSION

There is clear evidence of a workforce of public servants with CMSDs affecting multiple body regions.^12^ The significance of this burden is reflected in the substantial literature on the topic. A PubMed search for 2019-2024 yields 481 articles using the descriptors “quality of life,” “musculoskeletal disorders,” and “workers,” and 45 articles using “quality of life,” “musculoskeletal disorders,” and “public servants.” Among these, a 2021 study associated with Science reported a high prevalence of CMSDs - 74% of workers reported at least 1 symptom - with the lumbar, cervical, and wrist regions most frequently affected; women had a roughly 1.5-fold higher risk of developing such disorders.^13^ These findings align with the present results. Additionally, data from the Fundação Jorge Duprat Figueiredo de Segurança e Medicina do Trabalho document approximately 39,000 work absences in Brazil due to CMSDs, underscoring the public health and socioeconomic impact.^6^

Two sociodemographic factors frequently linked in literature to the pattern of illness in this population are age and sex. Studies^12^,^13^ corroborate that age is associated with locomotor system disorders, particularly around 45 years. Even so, the population analyzed here shows earlier onset - before age 40.

A Scandinavian study of office workers related early illness to continuous sedentary time (> 90 minutes without breaks), poor posture, and screen time > 6 hours/day.^14^ The present study aligns with the screen-time finding and adds sitting time as a related factor for CMSDs.

Regarding sex, our study identifies female sex as a predictor for the development of CMSDs. Literature-cited explanations include multiple workloads^15^ and menopause.^16^,^17^ In Brazil, menopause typically occurs between ages 45 and 55,^16^ a period marked by declines in muscle strength and hormonal changes associated with high rates of musculoskeletal discomfort.^17^ This raises the question of why women younger than this range already show such illness, beyond factors such as screen time, sitting time, and sedentary behavior.

From a behavioral standpoint, prolonged screen time and sitting appear to be risk factors in this population for both CMSDs and mental disorders.^18^ Extended sitting, repetitive tasks, limited movement, improper posture, lack of breaks during the workday, and spinal overload from static posture contribute to injuries and pain,^19^ reduce energy expenditure, and promote spinal curvature that impairs the digestive and respiratory systems and overloads back muscles.^20^ When combined with sedentary behavior, these factors also increase the risk of venous insufficiency.^21^

Beyond overuse of the musculoskeletal system and inadequate recovery,^20^ CMSDs also arise from a mismatch between job demands and individual functional capacity. Other ergonomics dimensions - not assessed in this study - warrant attention: physical (posture and movement adjustments), cognitive (optimization of mental processes and human-technology interaction), and organizational (workplace well-being and efficiency). In office settings, insufficiently structured ergonomics create suboptimal conditions that contribute to CMSD symptoms, as reported in the literature.^17^,^22^

As public servants at a federal university, participants perform bureaucracy-heavy tasks - document processing, minutes, committee work, procurement workflows - that require frequent computer use. Procurement processes for information technology equipment, desks, and chairs should specify adjustability, at minimum in depth and height, to support better body positioning throughout the workday.

Consistent with prior research, multiregional involvement predominated in this population, with an average of four affected regions.^23^ Similar work^23^ also reported an average of three affected regions, most often lumbar, cervical, and wrist-hand. These patterns resemble findings among blue-collar health-sector workers.^24^

Disorders mainly involved the upper body, as commonly described.^10^,^11^,^23^ Whether in the upper or lower trunk (and corresponding limbs), CMSDs are frequently driven by musculoskeletal wear^7^ and work factors,^5^ leading to pain, reduced mobility, diminished muscle strength, and higher risk of additional injuries.^12^,^22^ Beyond physical harm, CMSDs also affect mental health, contributing to anxiety, stress, and sleep impairment due to persistent pain.^9^,^17^,^18^ Institutionally, they are linked to reduced productivity through efficiency limitations, increased absenteeism, and greater medical costs.^3^,^6^,^12^

Multiregional pain management requires an initial evaluation of symptom etiology and a tailored pain-management strategy. The approach should incorporate patient expectations, ongoing assessment by a multidisciplinary team, health-education actions,^25^ nonpharmacologic therapies (eg, physical therapy, exercise, stretching, psychological therapies, and integrative health modalities),^26^ and pharmacologic treatment.^27^,^28^ Although further research is needed on their effectiveness,^29^ neuromodulation-based approaches are increasingly being considered for chronic symptom control.

Because of our initial hypothesis - that CMSDs affect QoL - and recognizing QoL as an individual’s perception of their social context and relationship to goals, expectations, standards, and concerns across spiritual, physical, mental, psychological, and emotional dimensions,^17^,^28^ we found that symptoms in all assessed body regions affected QoL to some extent, with the exception of the psychological domain.

Several body regions - particularly those in the upper trunk - had the strongest negative impact on QoL. These findings differ from another study^28^ reporting that pain, regardless of region or number of sites, impairs QoL. Studies assessing symptom intensity may help clarify factors that modulate this effect. Despite symptom presence, respondents’ QoL scores remained above 64%. This suggests that characteristics such as education, life stage, marital status, income, access to health care, and broader life context may be acting as protective factors.

In sum, the study population fits the profile of a public health problem related to CMSDs,^16^,^19^ with downstream consequences for workers’ QoL as well as negative impacts on employers and on health and social security systems.

At the national level, Brazil promotes workplace health education through tools aimed at managing and preventing occupational stress and conditions arising from non-ergonomic environments (physical, cognitive, and organizational), while empowering workers.^21^,^29^ Ongoing workforce training to prevent physical and emotional disorders can include lectures, discussion groups, psychological support, and workshops on mental health and communication.^21^

Brazil also recognizes February 28 as the National Day to Combat RSI/WRMD. In addition, the National Worker Health Policy established guidelines and strategies to ensure comprehensive health care for this group.^30^,^31^ We hope these policy measures are implemented and that workers have access to information about unhealthy workplace conditions, enabling them to collectively develop measures to mitigate these risks.

Limitations of this study include a potential healthy worker effect - where only workers with symptoms were inclined to participate - and the use of instruments that, although validated, capture respondents’ self-perceptions.

## CONCLUSIONS

Most civil servants reported chronic, multiregional CMSDs. Even so, global QoL remained high overall - except when wrist-hand symptoms were present. Wrist-hand symptoms were also associated with lower scores in the physical and environmental domains, while lumbar symptoms were linked to lower scores in the social domain. Weekly screen time, weekly time spent sitting, and female sex were the only variables positively correlated with the presence of CMSDs. Future research should include objective assessments of the workplace - addressing the three ergonomics dimensions - and longitudinal follow-up of workers. It may also be useful to examine variables that help explain the coexistence of CMSD symptoms and comparatively high QoL scores, such as whether women are in the climacteric or menopausal transition, to better understand female morbidity. Overall, these findings underscore the need for personal, collective, and organizational actions to improve workers’ health and QoL, particularly because of the early onset and multiregional nature of CMSDs in this population.
